# Modeling intrinsic potential for beaver (*Castor canadensis*) habitat to inform restoration and climate change adaptation

**DOI:** 10.1371/journal.pone.0192538

**Published:** 2018-02-28

**Authors:** Benjamin J. Dittbrenner, Michael M. Pollock, Jason W. Schilling, Julian D. Olden, Joshua J. Lawler, Christian E. Torgersen

**Affiliations:** 1 School of Environmental and Forest Sciences, University of Washington, Seattle, Washington, United States of America; 2 National Oceanic and Atmospheric Administration – Northwest Fisheries Science Center, Seattle, Washington, United States of America; 3 Tulalip Tribes Natural Resources, Tulalip, Washington, United States of America; 4 School of Aquatic and Fishery Sciences, University of Washington, Seattle, Washington, United States of America; 5 U.S. Geological Survey, Forest and Rangeland Ecosystem Science Center, Cascadia Field Station, University of Washington, Seattle, Washington, United States of America; University of Minnesota, UNITED STATES

## Abstract

Through their dam-building activities and subsequent water storage, beaver have the potential to restore riparian ecosystems and offset some of the predicted effects of climate change by modulating streamflow. Thus, it is not surprising that reintroducing beaver to watersheds from which they have been extirpated is an often-used restoration and climate-adaptation strategy. Identifying sites for reintroduction, however, requires detailed information about habitat factors—information that is not often available at broad spatial scales. Here we explore the potential for beaver relocation throughout the Snohomish River Basin in Washington, USA with a model that identifies some of the basic building blocks of beaver habitat suitability and does so by relying solely on remotely sensed data. More specifically, we developed a generalized intrinsic potential model that draws on remotely sensed measures of stream gradient, stream width, and valley width to identify where beaver could become established if suitable vegetation were to be present. Thus, the model serves as a preliminary screening tool that can be applied over relatively large extents. We applied the model to 5,019 stream km and assessed the ability of the model to correctly predict beaver habitat by surveying for beavers in 352 stream reaches. To further assess the potential for relocation, we assessed land ownership, use, and land cover in the landscape surrounding stream reaches with varying levels of intrinsic potential. Model results showed that 33% of streams had moderate or high intrinsic potential for beaver habitat. We found that no site that was classified as having low intrinsic potential had any sign of beavers and that beaver were absent from nearly three quarters of potentially suitable sites, indicating that there are factors preventing the local population from occupying these areas. Of the riparian areas around streams with high intrinsic potential for beaver, 38% are on public lands and 17% are on large tracts of privately-owned timber land. Thus, although there are a large number of areas that could be suitable for relocation and restoration using beavers, current land use patterns may substantially limit feasibility in these areas.

## Introduction

North American beaver (*Castor canadensis*) have long been recognized as ecosystem engineers, creating diverse and resilient wetland and riverine systems [1, 2]. Prior to near extirpation in the early 1900s due to over-trapping and habitat conversion [3], beavers and beaver-created wetland complexes were a ubiquitous component of riparian systems [4]. Many species depend upon these systems due to the high geomorphic complexity, aquatic thermal variability, and habitat diversity that they aford. For example, the decline in populations of some aquatic species, including Pacific Coho salmon (*Oncorhynchus kisutch*), have been partially attributed to the loss of beaver ponds [5], a feature that salmonids have evolved with since at least the Pleistocene [6].

Because of their abilities to modify streams and floodplains, beavers have the potential to play a critical role in shaping how riparian and stream ecosystems respond to climate change. The Pacific Northwest of the United States is experiencing increases in annual air temperature and decreases in snow pack and summer precipitation [7, 8], resulting in lower base flows, particularly in streams that rely on late season snowmelt. Climate shifts have altered stream-temperature regimes to the detriment of cold-water fishes, including Pacific salmon [7]. Recent increases in winter precipitation and storm magnitude have increased the potential for stream scour, channel incision, and floodplain disconnection, thereby promoting the drying of adjacent riparian areas [9, 10].

By damming streams, beavers create pond and wetland complexes that increase spatial heterogeneity and geomorphic complexity, species and habitat diversity, and therefore ecosystem resilience to climate-induced environmental change [11–13]. Beaver impoundments slow stream velocity allowing sediment suspended in the water column to settle, aggrading incised stream systems, and reconnecting streams with their floodplains [9]. The increase in surface water promotes groundwater recharge, storage, and supplementation during base flows [13]. The increased geomorphic complexity also promotes higher thermal variability and cold-water refugia in deeper waters and in areas of downstream upwelling.

Since the 1940s, beaver populations have begun to rebound in many areas of their historical range, and recolonize formerly occupied areas [14–16]. As they have done so, there have been responses in riparian ecosystem resilience and functionality [17].

Understanding and predicting where suitable beaver habitat exists within their geographic range can help inform recovery efforts, restoration planning, and conflict avoidance in populated areas. Beavers are generalist species [18] and can be found in most biomes of North America [19]. Regional habitat suitability index (HSI) models have been developed throughout North America to map suitable beaver habitat characteristics. These models predict *currently* suitable beaver habitat, but have less utility for predicting where beaver *could* be if they modify the landscape, or appropriate restoration actions or land-use management actions were taken. Because vegetation often does not meet criteria that a traditional HSI model would identify as suitable, many potentially suitable areas are not considered for restoration planning or relocation actions. Additionally, and perhaps more importantly, vegetation data are not currently mapped at fine enough spatial resolutions to allow for landscape-scale HSI models to be applied over larger spatial extents.

Intrinsic potential models provide an alternative to HSI models (which generally use both intrinsic and extrinsic predictors) by using geomorphic variables that are less prone to change through time. Intrinsic potential models have been previously used to inform fish habitat restoration work [20], and some have proposed using intrinsic potential for non-fish species, including beaver [21]. To date, however, beaver intrinsic potential (BIP) models have not been developed and field-verified. BIP models may be more appropriate than HSI models for predicting where beavers can likely exist within a watershed given the ability of beavers to modify variable habitat characteristics such as vegetation density and type. Intrinsic variables appropriate for use in BIP models are those that cannot be readily altered by beaver colonization. These include site features such as regional climate, precipitation regime, stream gradient, stream width, and valley width. The variables used in previous HSI models are often good predictors of current or historical beaver presence. However, they fail to identify areas that may become suitable if transformed by beavers into high quality habitat through restoration actions or management changes, and are therefore less useful in areas below carrying capacity or areas altered by anthropogenic impacts.

Here, we develop and apply a beaver intrinsic potential model that can predict where high quality beaver habitat currently exists and where colonization will occur as population levels increase or if management changes are made (e.g., expansion of a riparian buffer or greater implementation of non-lethal beaver management options). We developed our BIP model within the Snohomish River Basin, Washington State (USA), and validated it within the Skykomish River sub-basin. Our study had four primary objectives. The first objective was to develop a BIP model parameterized with readily available, remotely sensed public data, thus facilitating the transferability of the approach to other regions. The second objective was to evaluate the effectiveness of our BIP model within a large basin that has high levels of hydrogeomorphic complexity and highly variable beaver population densities. The third objective was to assess the potential for continued population expansion by assessing the degree to which beaver occupied areas with high intrinsic potential. Finally, we aimed to explore the potential constraints or barriers to colonization and occupancy of areas with high intrinsic potential by assessing land use in riparian areas with high intrinsic potential.

## Materials and methods

### Site description

**Fig 1:**
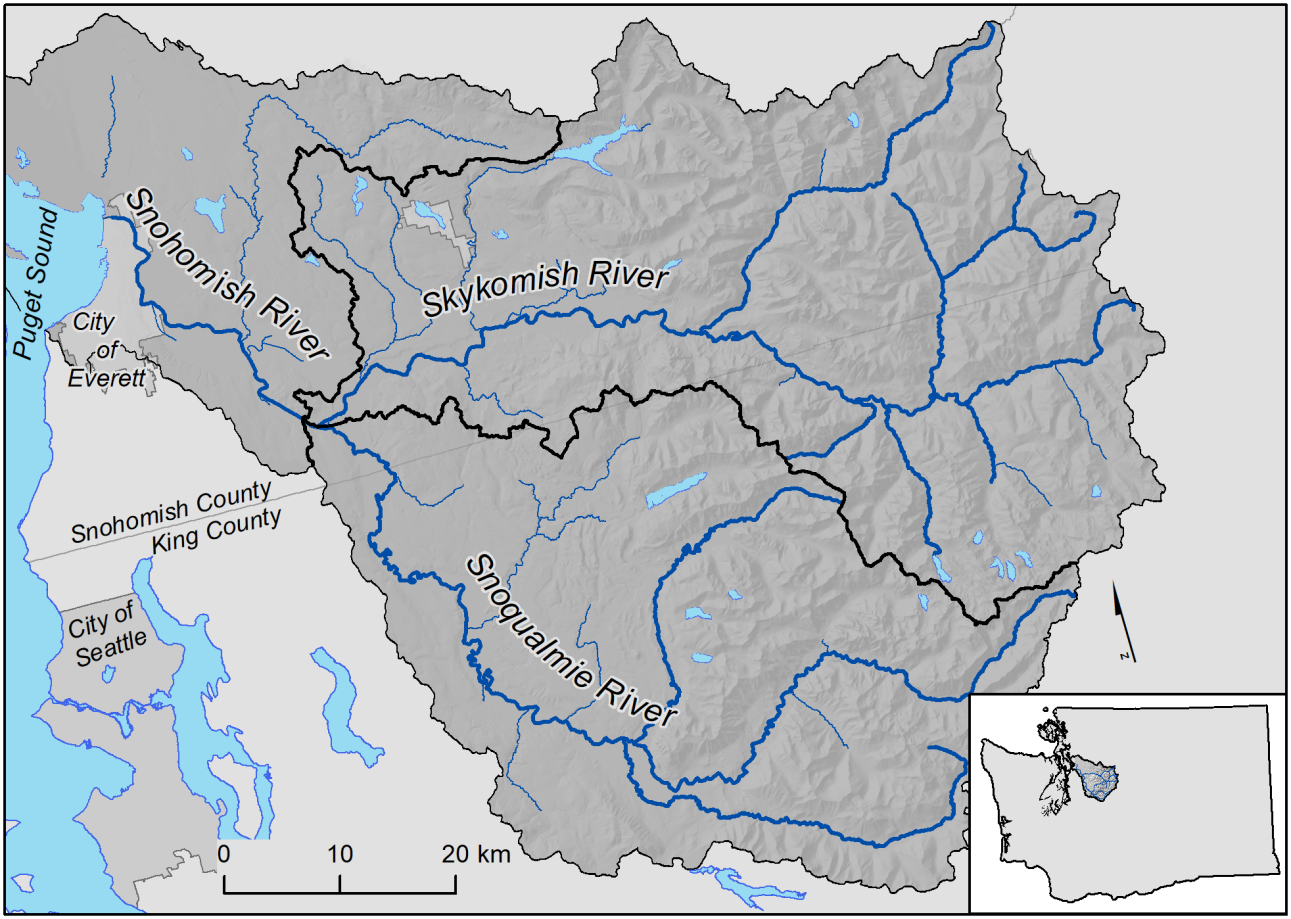
The Snohomish River Basin, Washington State (USA), showing the Skykomish River sub-basin, with major and minor river bodies. BIP was modeled in the entire Snohomish Basin and validated using survey data from the Skykomish sub-basin. Input data to inform model variables was derived from stream segments in the Snohomish watershed, but excluded the Skykomish sub-basin where model validation occurred.

The Snohomish River Basin (4,807 km^2^), located on the west slope of the Cascade Range in Washington State, was selected for BIP model development, and the Skykomish River sub-basin (2,160 km^2^) was used for model validation (Fig 1). The Snohomish basin was chosen because this area provides an excellent test case as the region’s hydrology has both high spatial and temporal variability; and abundant precipitation in the winter and sporadic precipitation in the summer add complexity to model development. Channel gradient and morphology vary greatly throughout the basin. Mountainous areas contain narrow, glacially carved valleys and high-gradient streams, which transition to low-gradient streams across a wide, hilly plateau and a large river floodplain with extensive side channels and tributary junctions. This varying geomorphic context allows for a more thorough assessment of geomorphic conditions (e.g., gradient and valley width) as suitability predictors. Additionally, the Snohomish Basin is a priority area for regional salmonid recovery work as well as a focus area for regional climate-change research, environmental monitoring, and intensive beaver population surveys [8,22,23]. The basin is representative of other watersheds in the region in terms of habitat conditions, importance for regional aquatic species, and potential climate refugia for wildlife [23–25].

### Data collection and model development

In preparation for BIP model development, we evaluated previously proposed beaver habitat suitability models documented in the literature (Table 1). The environmental variables most commonly cited as the best predictors of habitat suitability were vegetation composition, stream gradient, stream bankfull width, and stream valley width.

**Table 1:** Summary of general and regional beaver habitat suitability models identifying important environmental variables for predicting potential beaver occupation. Note that some studies focused on specific variable categories (e.g., vegetation) for the purpose of their study objectives.

Habitat Quality Variables	Allen	Retzer	Howard & Larson	Barnes & Mallik	Suzuki & Mc-Comb	Mc-Comb et al.	Pollock et al.	Cox & Nelson	Anderson & Bonner	Mac-farlane et al.	All Studies
	[26]	[27]	[28]	[29]	[30]	[31]	[5]	[32]	[33]	[34]	
Focus area: state/region	USA	Rocky Mts	MA	ON	OR	OR	WA	IL	WV	UT	n = 10
***Intrinsic***											**27**
Valley width		X			X		X	X			4
Stream length	X										1
Stream gradient	X		X		X	X	X	X	X	X	8
Stream depth & width			X		X	X	X			X	5
Stream bank steepness		X									1
Stream substrate						X					1
Stream power/flood risk		X		X			X			X	4
Basin size, perennial flow			X				X			X	3
***Extrinsic***											**17**
Vegetation composition	X		X			X			X	X	5
Vegetation density		X			X			X			3
Canopy cover	X			X	X	X					4
Canopy height	X										1
Stem diameter	X										1
Habitat & veg. area	X									X	2
Shoreline development ratio	X										1

Stream gradient is frequently correlated with beaver presence and is an ideal indicator of intrinsic potential due to its low likelihood to change over time. Beavers will most often colonize streams with gradients from 0 to 6% [26], although those below 3% are preferred [27]. Stream gradient is associated with a number of related site characteristics that make it a good predictor of suitable beaver habitat. Low gradient reaches have slower- moving water with finer substrates, which allows beavers to anchor dams to the stream-bed and provides mud for dam and lodge construction. Low gradient reaches also allow constructed dams to spread water across a larger area, increasing the surface area-to-dam ratio and decreasing costs and risks of dam-building (e.g., effort required for tree cutting and increased predation while on land) [9].

Stream bankfull width and associated environmental variables such as upstream contributing basin size and stream power have been identified as primary characteristics of potentially suitable habitat. Suzuki and McComb [30] reported that beavers preferred streams 3–4 m wide for damming with an outside range of 2–10 m in Oregon’s Coast Range, USA. Barnes and Mallik [29] found that the upstream watershed area was most useful for differentiating active and previously colonized sites from sites with no dams, in Ontario, Canada. Pollock et al. [5] analyzed the effect of stream power, a measure of stream force incorporating discharge and channel slope, and found that beaver dams were limited to sites with a stream power of less than 2,000 J*s^-1^*m^-1^ in streams of Washington State. Streams of a larger size or power have the likelihood of breaching dams during yearly high flow periods, so beaver preference for lower-power flows would be a successful adaptation strategy.

Valley width is a measure of stream confinement commonly used in HSI models and is often correlated with stream order and gradient. Earlier studies of habitat suitability did not use this metric as it requires more advanced spatial analysis software to generate basin-wide quantitative measures. More recent studies have found valley width to be a strong predictor of habitat suitability, and potentially for intrinsic potential [32]. This metric may be more important in mountainous and topographically diverse areas where stream confinement more frequently occurs [35]. Reaches with valley widths greater than 46 m were found to be optimal [26,36]. This measure, however, is likely regionally variable and requires further characterization [35].

We did not include extrinsic or modifiable factors such as vegetative cover in our model because those variables may change over time and are more difficult to assess remotely. Thus, the BIP model is intended to identify sites where the hydrogeomorphic, or underlying intrinsic physical conditions, are suitable for beaver dams. Unlike most habitat suitability models, the BIP model does not classify sites as unsuitable if habitat restoration, management changes, or beaver modification could allow beaver to thrive there.

We compiled and derived remotely sensed spatial, hydrogeomorphic, and other physical data for the Snohomish River Basin. We processed all spatial data layers and compiled them in a geographic information system (GIS) using ArcGIS. We obtained hydrography data layers consisting of a combination of field-verified and digital elevation model (DEM)-derived stream segments from King and Snohomish Counties, Washington. We derived stream slope, bankfull width, discharge, and stream segment breaks using the methodology outlined by Davies et al. [37]. The valley width for each stream segment was then calculated using the methodology described in Beechie and Imaki [38]. Valley width was defined as the average width of the area adjacent to a stream segment that was within 2 m vertical elevation of the channel elevation. We obtained soil type and permeability layers within the study watershed from U.S. Forest Service soil inventories [39]. Soil types (e.g., sandy-loam) were converted to percent silt, clay, and sand so that these data could be treated as continuous variables instead of factors for multivariate analysis during validation.

**Fig 2:**
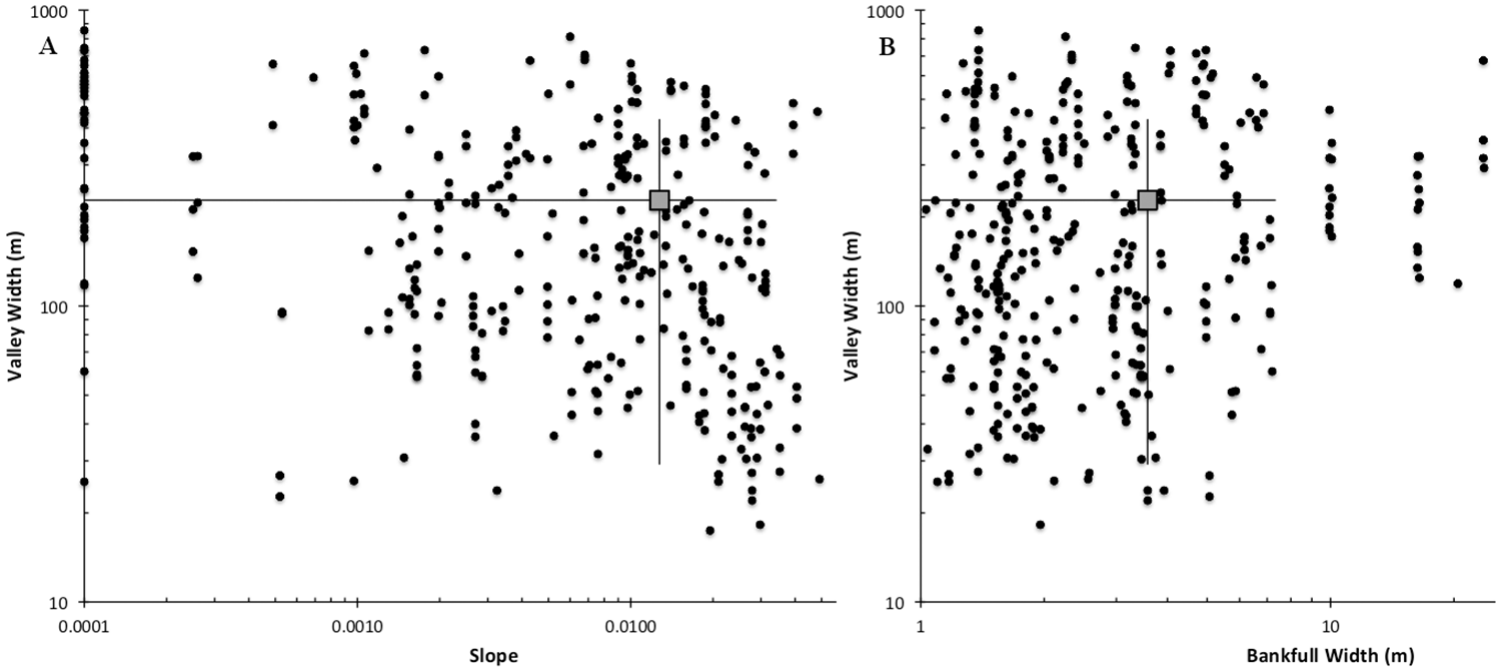
Geomorphic characteristics of stream segments in the Snohomish River Basin occupied by beaver. (A) Valley widths versus slope, (B) valley width v. bankfull width. Crosshair lines represent the standard deviations, and square symbols are the means. Note that the scale is logarithmic. The data show that dam-building beaver generally prefer streams with percent slope < 0.04, bankfull width < 8 m, and valley width > 30 m.

To assist in identifying the range of intrinsic potential habitat within our study basin, we selected 501 stream segments showing signs of current or recently abandoned beaver ponding using Google Earth and U.S. National Agriculture Imagery Program (NAIP) orthographic imagery. These segments were sampled from the Snohomish watershed but excluded the Skykomish sub-basin where model validation occurred. We described stream slope, stream width, and valley width within each segment to identify the range of conditions present at sites that beavers colonized (Fig 2).

Based on the range of conditions present at potentially suitable sites, we assigned a ranked value from 0–4 to each of these variables, commensurate with their level of intrinsic potential according to the criteria in Table 2. Ranking values for each variable were based on a combination of expert opinion and analysis of habitat preference at locations identified in Fig 1. Higher weight (value 4) was given to metrics with high intrinsic habitat potential (e.g., slope ≤ 1%). We assigned a final BIP score for each segment by summing the ranked scores of stream slope, stream width, and valley width (analogous to the IP model of Burnett et al. [20]). We assigned intrinsic potential scores to all stream segments within the Snohomish River Basin to produce the BIP model. The model possesses four predictive categories of beaver intrinsic potential: No BIP, Low, Moderate, and High BIP, numbered 0–3, respectively.

**Table 2:** Additive scoring criteria for environmental variables in each stream segment used to categorize the beaver intrinsic potential (BIP) of all 5,182 km of stream segments in the Snohomish River Basin. Total BIP Score was found by adding the variable scores, Stream Slope + Stream Width + Valley Width (max = 12, min = 0), and adjusted to categories 0–3 for ease of display and analysis.

Stream slope & score			Stream width & score			Valley width & score			Cumulative score	Adjusted score	BIP categories
< 1%	4		< 7 m	4		> 30 m	4		11–12	3	High
< 2%	3		< 10 m	3		< 30 m	2		10–11	2	Med
< 4%	2	**+**	< 18 m	2	**+**	< 20 m	0	**=**	8–10	1	Low
< 6%	1		< 24 m	1					< 8	0	No BIP
< 10%	0.5		> 24 m	0							
> 10%	0										

Following completion of model development, we compared the spatial distribution of modeled intrinsic potential to land use in the Snohomish Basin to identify how habitat was distributed across the landscape and where it might be at odds with existing human use. Land use was identified within 30-m buffers of all modeled streams using Snohomish County zoning GIS data.

### Field validation of remote sensing based BIP

We field-verified the BIP model in the Skykomish River sub-basin, testing how well the modeled intrinsic potential predicted field-assessed intrinsic potential at surveyed sites. We limited survey locations to those that were within stream segments that had a relatively homogenous gradient and stream width within the mapped stream segment. Using a quasi-random approach, we selected 100 stream segments from each of the four BIP classes (0–3) for a total of 400 survey sites, which were reduced to 352 due to access limitations in some cases. We conducted a blind assessment of conditions at each field location during base flow conditions (i.e., July through September). In instances where there was uncertainty, we revisited the site during higher flow conditions. The same metrics used to construct model scores within mapped stream segment, stream slope, width, and valley width, were evaluated in the field. A field score for each segment was found using the same methods used in the model (Table 2). Together, these surveys evaluated 32.1 stream km.

Prior to validating model scores with field conditions, we established an intrinsic potential threshold, or a cutoff, separating high IP model scores from those thought to be less suitable using methods in [40]. We established a threshold between values 1 and 2, thereby grouping model values 0 and 1 as no BIP, and values 2 and 3 as high BIP. We arrived at this threshold by comparing stepwise combinations of model scores.

We performed a validation test for the BIP model’s ability to predict the site’s intrinsic habitat potential. Validation was conducted by comparing modeled suitability with observed BIP at 352 stream segments using a contingency table. Overall model accuracy, sensitivity, and specificity were assessed.

### Predicting beaver occupancy

In addition to comparing our model predictions to BIP assessed in the field, we determined the degree to which BIP based on remotely sensed data predicted beaver occupancy. We surveyed the same 352 sites used in our validation effort for signs of both current and historical occupation. Site occupancy was determined by the presence of recent beaver sign, such as freshly chewed sticks, cut logs, fresh scent mounds, or presence of actively maintained dam or lodge structures (following Snodgrass and Meffe [41]). In areas with questionable occupancy, we confirmed presence with multiple revisits, wildlife cameras, and by placing small notches in the crest of dams and monitoring them through the summer for repair. A number of sites appeared to be recently vacated yet retained high intrinsic habitat potential; these sites lacked new beaver sign or recently maintained structures, but these sites still had dams that impounded water to near the crest, supporting a large wetland complex.

## Results

### Data & model development

**Fig 3:**
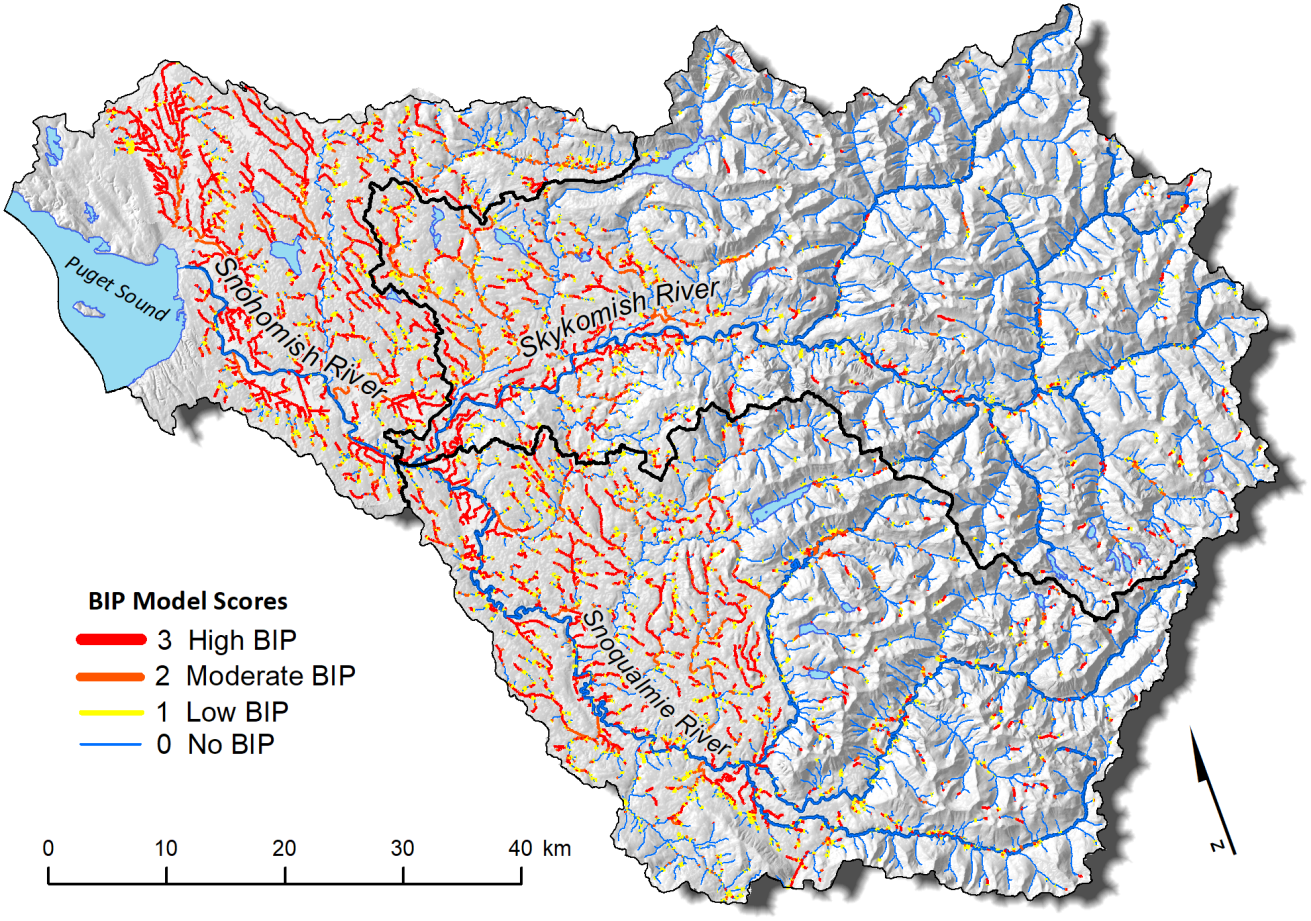
BIP model in the Snohomish Basin. Large, low-gradient rivers and small, high-gradient streams are grouped as having low or no BIP, while small to mediums sized low-gradient streams have moderate or high BIP.

The BIP model assigned one of four BIP values (*High*, *Moderate*, *Low*, or *No BIP*) to 48,397 stream segments comprising 5,019 stream km within the Snohomish River Basin (Fig 3 and Table 3). Most reaches with high BIP were concentrated in lower gradient areas outside of the Cascade Mountains. Approximately 23% of all streams were categorized as high BIP, 10% as moderate, and 8% as low BIP. The remainder of the streams (~60%) in the basin were classified as having no intrinsic potential for beaver colonization. Most of these were high gradient headwaters. The majority of high BIP reaches were located in low gradient streams (i.e., ≤ 3%) with wide floodplains or on side channels of large rivers. Smaller pockets of high BIP reaches were also identified in more mountainous areas.

**Table 3:** BIP model predictions for beaver habitat intrinsic potential in the Snohomish River Basin, showing number and total length of streams segments by category. Observed conditions, number of validation sites visited within each modeled BIP class, and beaver presence is described for field-validated sites, which occurred within the Skykomish River subbasin.

Modeled Conditions				Observed Conditions			
BIP	Stream segments	Length (km)	% of stream segments	Validation sites	High BIP sites	Current & historical occupancy	Occupied
High	11,768	1,171	23%	91	87	26	34
Moderate	4,987	481	10%	82	57	59	5
Low	5,058	389	8%	83	1	0	0
No BIP	26,584	2,978	59%	96	0	0	0
Total	48,397	5,019	100%	352	145	85	39

### Field validation and beaver occupancy

**Fig 4:**
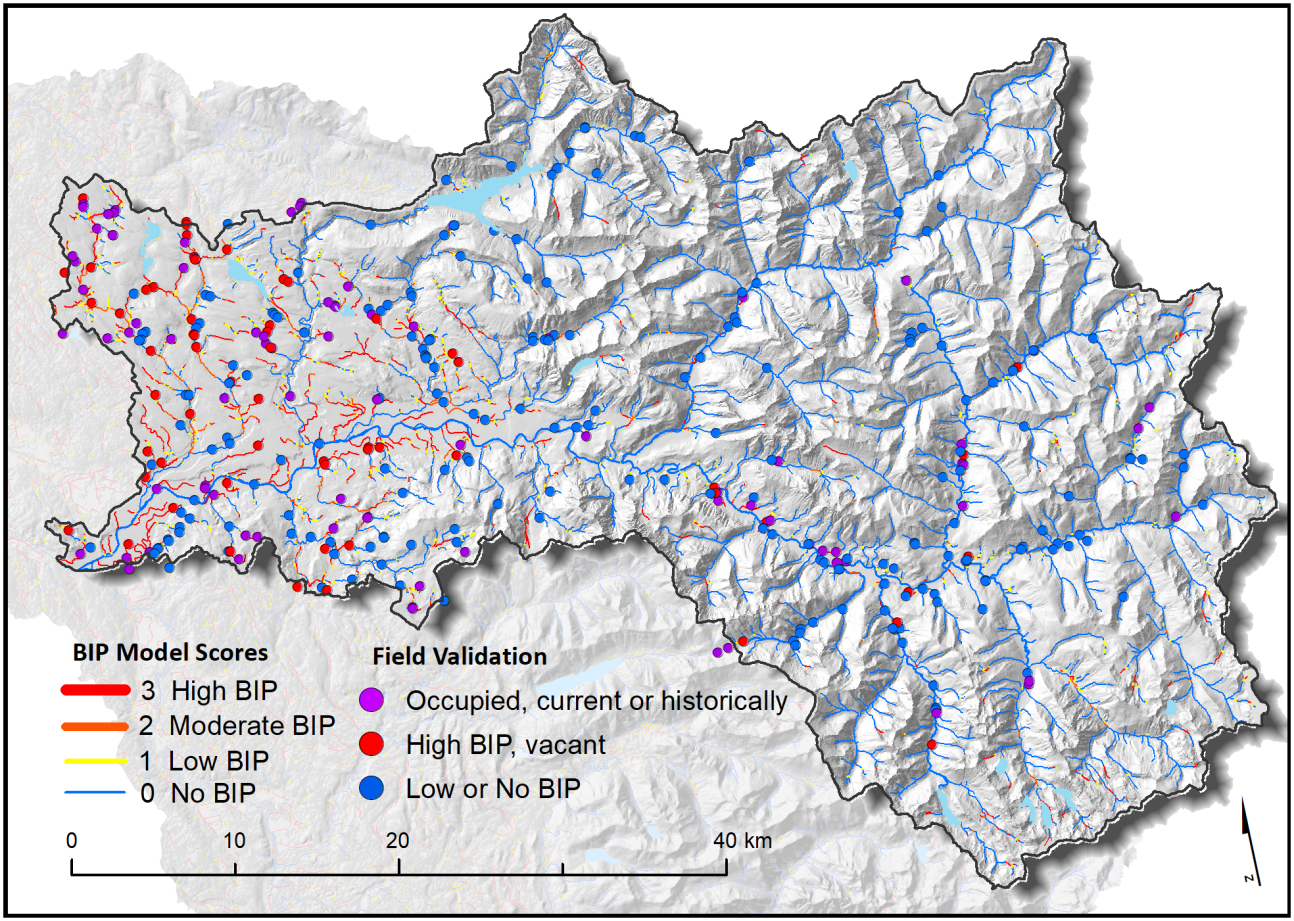
Field validation sites in the Skykomish subbasin.

Within 352 randomly selected field points, we rated 91 segments modeled as high BIP, 82 segments modeled as moderate BIP, 83 low BIP segments, and 96 modeled as no BIP (Fig 4). Comparison of modeled (expected) site scores with field-observed site conditions revealed high degrees of accuracy, specificity (i.e., rate of low BIP prediction accuracy), and sensitivity (i.e., rate of high BIP prediction accuracy) (>85%) (Table 4). Error in stream alignment in the original stream data layer was likely the greatest source of model error, and when present, likely precipitated other environmental variable error. Of the 352 stream segments that were field verified, 39 sites were actively colonized and 46 appeared to be currently vacant but had evidence of historical dam-building (Table 3). Fifty nine percent of all sites classified as having moderate or high intrinsic potential had signs of current or past beaver occupancy. No site that was classified as having low intrinsic potential had any sign of beavers (Table 4).

**Table 4:** Model validation contingency table and supporting test statistics, comparing field-observed BIP with modeled BIP (left), and evaluation of model prediction at sites currently or historically occupied by beavers (center), and actively occupied sites (right).

	Field-observed BIP		Evidence of beaver	
BIP model prediction	Low	High	Current or historically occupied	Currently occupied
No & low	177	1	0	0
Moderate & high	30	144	85	39
Accuracy	0.92		0.99	0.99
95% CI	(0.88, 0.94)		(0.96, 0.99)	(0.96, 0.99)
Sensitivity	0.99		0.99	0.99
Specificity	0.86		0.99	0.99
Observations	352		85	39

### Land use

**Fig 5:**
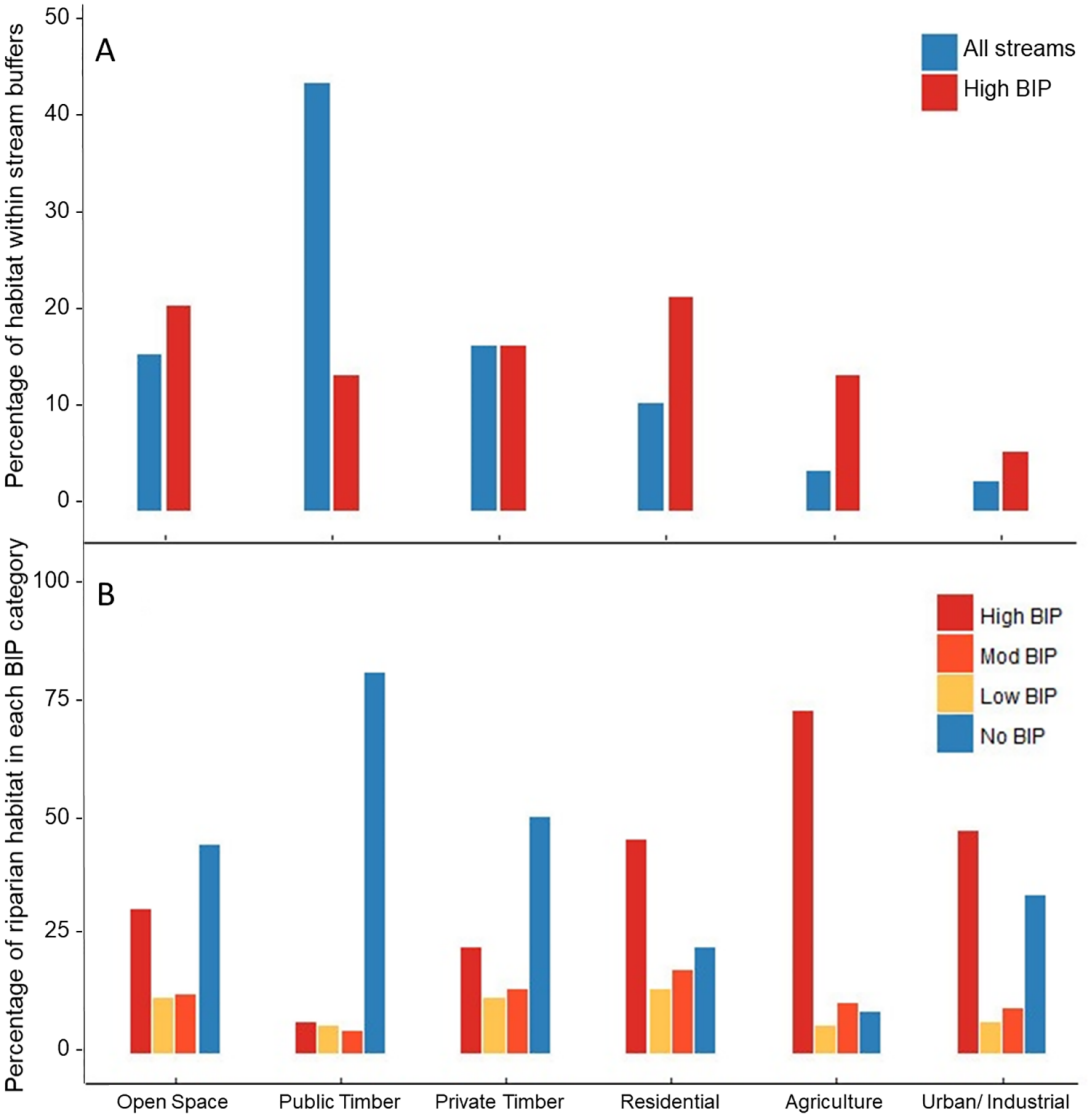
(A) Distribution of 30-m stream buffers and high quality BIP segments in the Snohomish Basin by land use type. (B) Proportion of high, moderate, low and no BIP by land use type.

Although 60% of all 30-m stream buffers were located within areas designated as open space in Washington State [42] and on public timber lands in the Snohomish Basin—areas likely to experience the lowest conflict from beaver colonization due to fewer competing human interests– 79% of these areas were classified as having no intrinsic potential for beavers. Over half of areas with high BIP (59%) were located in human-dominated landscapes, such as industrial, agricultural, residential, and privately held land use types (Fig 5A). Furthermore, higher intensity, human dominated land uses, such as agriculture and residential development had much greater proportions of higher quality BIP habitat than natural lands, demonstrating why human-beaver conflicts are so common (Fig 5B).

## Discussion

Beaver reintroduction and relocation hold much potential for habitat restoration and for addressing the impacts of climate change [43]. Identifying where to perform those introductions over large areas, however, remains a conservation challenge. Here, we demonstrate how models of remotely sensed intrinsic habitat potential identify potential habitat with high confidence. This approach offers a straightforward method for developing accurate estimates of the potential for beaver habitat using readily available data. The accuracy of this model makes it particularly useful for identifying sites that are suitable for beaver relocation and beaver-assisted restoration.

Beavers present a unique challenge and opportunity for accurate habitat model development, especially in areas where populations are below carrying capacity or where a large amount of unimproved vacant habitat exists. The use of BIP models has the advantage over traditional habitat suitability models of detecting potential habitat, regardless of the current vegetative cover or land use. In areas such as the Skykomish subbasin, where our model and intensive site surveys found population levels to be well below carrying capacity, the effects of beaver colonization (e.g., dam building, pond formation, and subsequent aggradation of stream channels [9]) have the potential to increase the suitability of surrounding habitat.

Our study demonstrates that there is a large amount of potential habitat within the Snohomish watershed that remains unoccupied by beavers, much of which is in fragmented landscapes and in ownership patterns to which it is not easy to apply beaver restoration (Fig 5). Although there appear to be many opportunities to use beaver as a restoration tool and to mitigate the effects of climate change throughout the Snohomish basin, many of these opportunities exists in areas where ownership patterns are diverse. Watersheds such as the Skykomish sub-basin, however, which are dominated by public ownership, provide ample opportunities to test how beavers can be reintroduced into landscapes where they are absent or at low population levels.

In the Skykomish sub-basin, site assessments showed no sign or evidence of past beaver presence in many stream segments categorized as geomorphologically suitable beaver habitat by the BIP model and site surveys. Field surveys indicate that approximately 75 percent of geomorphologically suitable sites in the basin are vacant, raising the question: why are there no colonies in these areas? A recent study of the European beaver (*Castor fibre)* demonstrates that in areas where beaver populations are depressed, but unexploited, they will increase rapidly towards carrying capacity [44]. It is possible that a combination of top-down, bottom-up, and abiotic controls are preventing colonization or suppressing population growth rates. Top-down and abiotic constraints on recovery rates could include high predation levels, environmental stressors (e.g., harsh winter conditions at higher elevations), limited dispersal corridors due to fragmented habitat and constrained topography, or undocumented recreational trapping. Bottom-up pressures may include previously unexplored interactions such as conifer encroachment into historical beaver meadows and a shift in vegetative composition to include less palatable species. Given these pressures, uncolonized areas meeting minimal habitat requirements may experience a very gradual expansion as beavers go through the initial steps of transforming each site’s morphology and vegetative composition. Areas surrounding currently occupied sites have potential for reintroduction and restoration because of their proximity to beaver population sources and the favorable ecological conditions created through intermittent colonization by beavers in the past. It is also possible that such reaches were inhabited by beavers prior to their extirpation by European trappers in the early to mid-1800s, but that the length of time passed since extirpation, land-use activities, and the humid climate has removed more obvious signs of their existence. Unfortunately, we could find no historical records characterizing pre-European beaver abundance in our study basin, so we have no such data to which we can compare our model results.

Like many watersheds, the Snohomish is projected to experience substantial hydrologic change over the next 100 years due to changing climatic conditions [45]. Summer precipitation is projected to decrease and winter precipitation to increase. This may convert some perennial streams to seasonal streams and in winter months may result in an increase in stream power. Beavers may be able to mitigate some of these hydrologic changes by reducing stream power, allowing reaches to aggrade [9] and converting higher gradient streams into stepped pools that disperse energy. These pools also impound surface water, allowing it to recharge groundwater [46] and supplement streams during low-flow periods [47].

The recolonization of large areas and the subsequent landscape-level changes that may ensue could result in changes to the hydrogeomorphic variables that BIP models use. Over time, effective stream gradient may change as beavers create series of step pools, and stream power may decrease as water is spread over larger areas. Additionally, as flow regimes change, so will some of the physical and hydrologic characteristics of streams, including stream size and power. Areas experiencing hydrologic and hydrogeomorphic changes due to either climate change or rapid population growth, respectively, may require the model to be run with updated climate and flow data.

Like any model, the intrinsic potential model described here has its limitations. For example, our BIP model captured most riverine wetlands but missed adjacent palustrine wetlands (*sensu* [48]). During field surveys, crews often discovered depressional wetland habitat located adjacent to, but separate from streams. Where depressional wetlands share the subsurface hydrology of the adjacent stream, there is potential for beavers to expand this habitat substantially creating stream-wetland matrices. These areas can enhance surface and groundwater storage, provide periodic surface connections to backwater rearing areas for juvenile fish, and provide unique habitat for amphibians and other riparian species [49,50]. While this model may be experimentally applied to areas outside of the Puget Sound region, the scope of inference for this model is intended for mesic watersheds of the Cascades and Puget Sound lowlands. It is likely that the underlying variables comprising our model—stream gradient, stream width, and valley width—will be important in most areas. Additional factors may dictate BIP in other regions as demonstrated in Table 1. Another potential limitation of our model is the spatial quality of the remotely sensed data which may cause errors in the alignment of stream layers with other geospatial data layers. In spite of these potential limitations, using a BIP model such as the one described here can inform practitioners as they identify candidate sites for beaver relocation without extensive field surveys or complex modeling.

## Supporting information

S1 Table Environmental variables within each stream segment used in BIP model construction. (TXT)
